# A Tester to Evaluate the Correct Placement of Earplugs

**DOI:** 10.3390/ijerph19148482

**Published:** 2022-07-11

**Authors:** Emil Kozlowski, Rafal Mlynski, Leszek Morzynski, Adam Swidzinski

**Affiliations:** Department of Vibroacoustic Hazards, Central Institute for Labor Protection—National Research Institute, Czerniakowska 16, 00-701 Warsaw, Poland; rmlynski@ciop.pl (R.M.); lmorzyns@ciop.pl (L.M.); adswi@ciop.pl (A.S.)

**Keywords:** hearing protection devices, earplugs, personal protective equipment, fit testing, sound attenuation, noise

## Abstract

The use of hearing protection devices is one possible way of reducing the negative impact of noise on hearing. However, it is important to keep in mind that only properly used hearing protection devices provide adequate hearing protection. The aim of this article is to describe a newly developed tester to verify the correct placement of earplugs in the ear canal. This tester was developed using easily accessible and low-cost components. It implements the real-ear attenuation at threshold (REAT) method by which the sound attenuation of hearing protection devices is determined. The headphones with a greater low-frequency attenuation value were selected for use in the tester. The results of the sound attenuation measurement performed with the use of the tester did not differ by more than 5 dB compared to the measurements performed with the use of the Norsonic NOR838 system dedicated to this purpose. The developed tester is considered to be a device that will obtain reliable sound attenuation values. Thus, it can also be used as a device with which the correct placement of earplugs in the ear canal can be assessed.

## 1. Introduction

Noise is one of the most frequent causes of hearing loss in adults. Worldwide, a total of 16% of people are estimated to have hearing loss associated with occupational noise [1]. However, it is possible to introduce some preventive measures that reduce exposure to this type of noise, resulting in a reduction in the risk of hearing loss. One such action is the use of hearing protection devices. At the same time, it is important to bear in mind that only the correct use of hearing protection devices can lead to the effective protection of hearing. The effectiveness of hearing protection devices against noise is mainly influenced by the sound attenuation of hearing protection devices, the correctness of their placement, and the consistency of their use [2]. Sound attenuation is a parameter of hearing protection devices that is independent of their user. In Europe, this is determined in accordance with the requirements of EN ISO 4869-1:2018 [3]. The other aspects related to the effectiveness of hearing protection devices are directly influenced by their user. For example, workers use worn or damaged hearing protection devices that are limited in effectiveness [4,5,6]. In a working environment, it is quite common for employees to use hearing protection devices and other personal protective equipment at the same time, resulting in leakages and the reduced effectiveness of the hearing protection devices [7,8,9,10,11,12,13]. Another aspect affecting the effectiveness of hearing protection devices is whether they are used at all times while in a noisy environment. Unfortunately, it is not a rare practice to interrupt the use of hearing protection devices or not to use them at all [14,15,16,17,18]. Interruptions in the use of hearing protection devices drastically affect their effectiveness [19]. Another reason for the reduced effectiveness of hearing protection devices is their improper placement due to a lack of knowledge as to how to use them correctly [20]. Research indicates that training is essential for improving the effectiveness of hearing protection devices [21,22,23,24,25,26]. Training on the correct use of hearing protection devices can be carried out in various ways. There are many induction training courses available in the form of drawings, photos, or videos. It is also good to train employees by having qualified people demonstrate how to properly use hearing protection devices. An additional solution to support the training and allow for checking the correctness of the placement of hearing protection devices is the use of devices designed specifically for this purpose [27,28,29,30,31,32]. These devices generate acoustic signals and based on the reaction of their users, it is possible to assess the correctness of the placement of hearing protection devices, in particular earplugs, where the correctness of their placement is a significant problem [13,33].

The purpose of this paper is to present the concept and implementation of a device used to assess the correct placement of earplugs in the ear canal, which is called a tester. The results of the tests verifying the operation of the device in question and tests leading to the selection of headphones with which the device is equipped are also presented.

## 2. Material and Methods

### 2.1. Concept and Design of the Tester

It was assumed that the tester under development would consist of easily accessible and low-cost components, that it would be an integral device without needing to be connected to other controlling devices (e.g., personal computer), and that it would employ a REAT measurement method by which it would be possible to determine the correct placement of earplugs in the ear canal. The REAT method is based on the measurement of the hearing threshold of subjects with (occluded) and without (unoccluded) hearing protection devices. To measure the hearing threshold, Békésy audiometry is used. The hearing threshold is determined by averaging 20 reversals for a particular frequency after rejecting the first four initial reversals. The measurement procedure includes the rejection of the result in the case of a significant discrepancy between the subject’s responses. The difference between hearing thresholds (occluded and unoccluded) is equivalent to the sound attenuation of hearing protection devices. A typical sound attenuation measurement is performed with 16 subjects. The sound attenuation results of individual subjects are used to calculate the mean value of the sound attenuation, the standard deviation, and the APV (assumed protection value) parameter, which is the difference between the mean value of the sound attenuation and the standard deviation. These parameters of hearing protection devices, as determined in accordance with the requirements of EN ISO 4869-1:2018 [3], are given in their user manual. Typically, sound attenuation measurements are carried out in a specially designed chamber providing suitable acoustic conditions with respect to the sound field, background noise, and reverberation time. The subject responds with the use of an external response button to a test signal that is pink noise filtered in 1/3 octave bands with a center frequency from 125 Hz to 8000 Hz. In contrast to the standard measurements in the designed device, the sound source will be headphones. Using a response button integrated into the device, the user will respond to the test signal so that the user’s hearing threshold with (occluded) and without (unoccluded) earplugs can be determined. On this basis, the sound attenuation of earplugs will be determined. Similar to the case of typical measurements, the test signal will be pink noise filtered in 1/3 octave bands with a center frequency from 125 Hz to 8000 Hz, and Békésy audiometry will be used.

The evaluation of the correct placement of earplugs using the tester will consist of comparing the sound attenuation values of the earplugs obtained by the tester with the sound attenuation data of these earplugs as specified in the user manual.

The design of the tester consists of the following components:

- Raspberry Pi Zero v.1.3 single-board computer;
- Liquid-crystal display (LCD) with a capacitive touch panel;
- Test signal generation system;
- Headphones;
- Response button;
- Power supply system.

**Figure 1:**
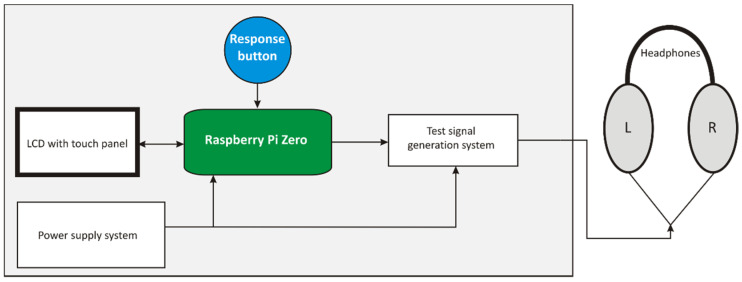
Basic flowchart of the tester.

The basic flowchart of the tester is shown in Figure 1.

The Raspberry Pi Zero v.1.3 single-board computer (RPi), commonly used for the implementation of embedded systems, will control the entire tester and at the same time, act as the test signal generator. The RPi is equipped with a 1 GHz processor and ARM11 core with 512 MB of random access memory. The RPi board is equipped with a micro secure digital (SD) memory card slot and a number of electronic connectors: power, a mini high-definition multimedia interface (HDMI), a universal serial bus (USB), and a 40-pin connector with a 2.54 mm standard (the so-called gold pin) for general purposes. The operating system and other data were stored on the supplied micro SD card. Additionally, the RPi board was provided with an extension module in the form of a ‘hut’ (printed circuit board attached to the computer board via a pin connector) constituting a USB hub. This hub allows for up to four USB devices to be connected to the RPi and is used to connect the touchpad of the LCD as well as to connect other devices necessary during the commissioning and testing of the tester design (e.g., keyboard).

Communication with the user is provided by a 1024 × 600 pixels 7-inch LCD connected with RPi with HDMI to transmit the image and a USB used as both a power connector and to transmit the information from the touch panel.

#### Test Signal Generation System

**Figure 2:**
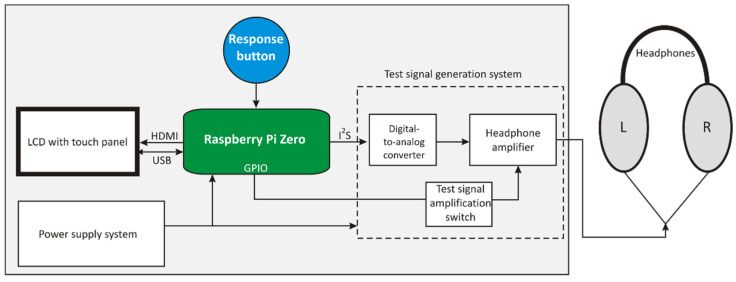
Modified flowchart of the tester in the solution using the digital-to-analog converter and headphone amplifier supplemented with a test signal amplification switch as the test signal generation system. GPIO: general-purpose input/output.

To avoid excessive load on the operating system used in the device, a 24-bit digital-to-analog converter (DAC), in the form of an extension hut for the RPi, was proposed. The DAC communicates with the RPi using the Inter-IC Sound (I^2^S) interface via a 40-pin connector. Since the I^2^S support system is one of the peripherals of the Broadcom BCM2835 processor on which the RPi was developed, and the drivers of this module in the operating system used are well-tuned, the digital transmission of the test signal exerts a low load on the processor and the remaining RPi resources. After conversion to the analog form, the test signal is amplified accordingly in the power amplifier. This solution uses the Topping NX1s high-quality headphone amplifier as a power amplifier with a signal-to-noise ratio (SNR) of no less than 117 dB. This amplifier allows the test signal to reach a higher sound pressure level than the sound card. The system tests also showed no distortions of the test signal. Additionally, this system makes it possible to step change the level of reinforcement in the power amplifier. This solution allows for an increase in the dynamics of the generated acoustic signal, in other words, it enables an acoustic test signal with lower (measurement without earplugs) and higher (measurement with earplugs) sound pressure level values to be generated for a digital signal with lower dynamics (i.e., it is not necessary to use small signals when generating a quiet test signal). Amplification in the headphone amplifier was increased by 12 dB when generating a test signal during the measurement with inserted earplugs. The modified flowchart of the tester considering the application of the DAC and the headphone amplifier configuration is shown in Figure 2.

### 2.2. Tester’s Operating Principles

**Figure 3:**
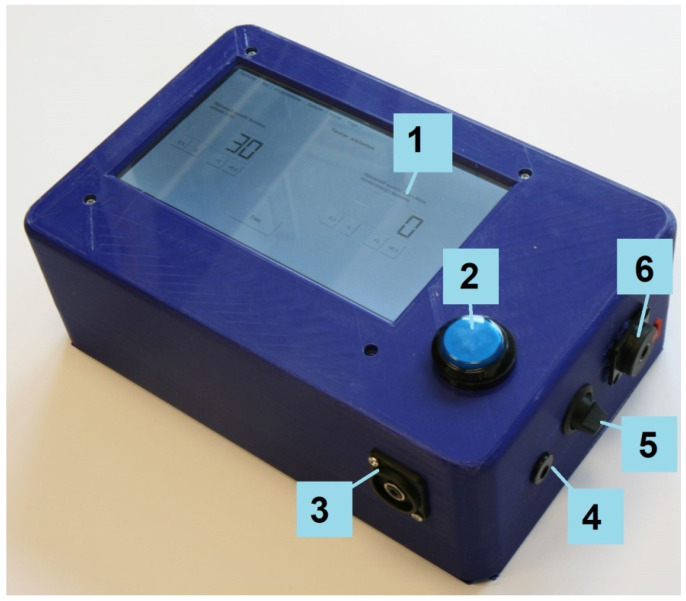
The implemented tester: Diagonal view (1. LCD with a touch panel, 2. response button, 3. headphone connector, 4. charging connector, 5. on/off switch, 6. 6.5 mm jack connector for an additional external response button added for testing purposes).

**Figure 4:**
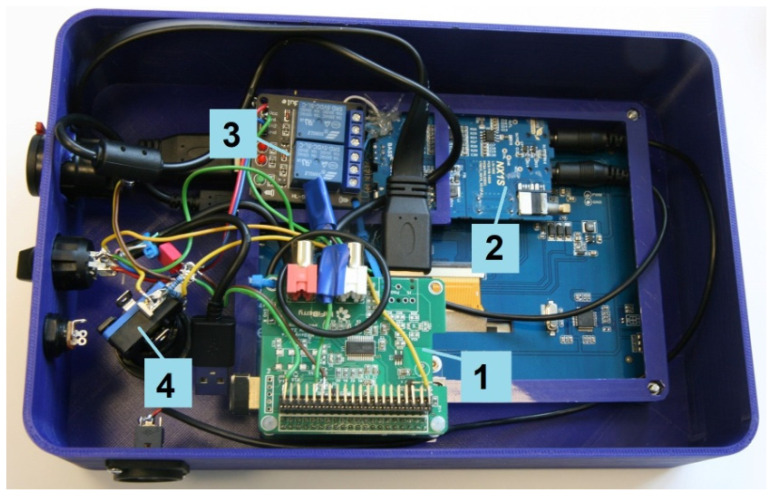
The implemented tester: The view of the electronic systems inside the housing (1. RPi with DAC, 2. headphone amplifier, 3. test signal amplification switch (0/12 dB), 4. response button).

**Figure 5:**
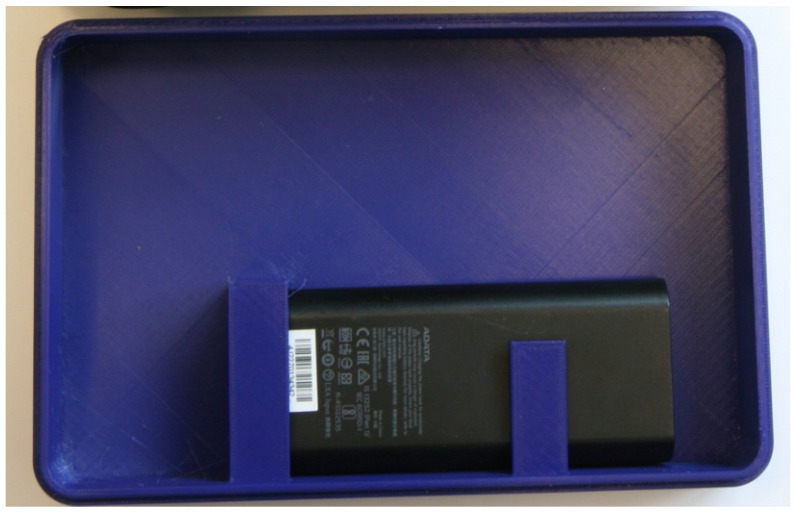
The implemented tester: Lower housing cover with the tester’s supply battery fitted.

In Figure 3, Figure 4 and Figure 5, the tester made according to the diagram in Figure 2 is presented. The electronic systems of the tester were installed into a polylactic acid housing printed on a 3D printer. A battery with a capacity of 12,500 mAh was used to power the tester. The battery was equipped with suitable protections, a charging system, a battery charge indicator, and a 5 V output. The 5 V voltage is sufficient to supply all the systems, apart from the amplifier. A step-down system to decrease the voltage from 5 V to 4.2 V was used to supply the amplifier. The tester operates under special control software dedicated to RPi built on Raspbian (LINUX). The tester’s control software was developed in C++. A file system monitor was used to handle the physical user response button. The Raspbian system’s directory contains a virtual folder where information about the physical state of the I/Os is stored in a text format. Monitoring the files allows for the generation of an interruption when a given file is changed. This makes it easy to detect when a response button is pressed.

### 2.3. Statistical Analysis

In order to compare the sound attenuation results obtained using the developed tester and the Norsonic NOR838 system for the measurements of sound attenuation, a statistical analysis was performed using a parametric Student’s *t*-test. The calculations were performed using MATLAB R2010b version 7.11.0.584 (MathWorks Inc., Natick, MA, USA).

### 2.4. Ethics and Bioethics Commission

Prior to the commencement of this research, an application for the study was submitted to the Ethics and Bioethics Commission of Cardinal Stefan Wyszyński University in Warsaw. The commission issued a positive review (No KEiB-20/2020) of the study, providing consent for the implementation and publication of the research results.

## 3. Results

### 3.1. Headphone Selection

Two headphone models were selected for potential use in the tester, Extreme Isolation EX-29 and Vic Firth SIH2. The manufacturers of these headphone models claim they have high insulation from external sounds (sound attenuation). The Extreme Isolation EX-29 headphones are available in two different versions, which slightly differ in their headband design and color. The headphone models under consideration are characterized by a wide frequency band of 20 Hz–20 kHz and a sensitivity of 114 dB/mW and 110 dB/mW at 1000 Hz for Extreme Isolation EX-29 and Vic Firth SIH2, respectively. These headphones are therefore capable of reproducing a test signal with an appropriate frequency and sound pressure level to enable the measurement of the hearing threshold both in the absence and during the use of earplugs. Additionally, the headphones were tested to verify their sound attenuation, which is important when the tester is used in a room with insufficiently favorable acoustic conditions (i.e., when there is a relatively high level of background noise.

The measurements of the sound attenuation of headphones were performed with the use of Békésy audiometry in a room designed for testing hearing protectors in accordance with the requirements of EN ISO 4869-1:2018 [3] with a limit of four subjects (three men and one woman between the ages of 20 and 45). As mentioned earlier, sound attenuation measurements of hearing protectors are conducted with 16 subjects. The number of subjects in this study was limited to four for two reasons. The first reason was the comparative nature of the study (i.e., the purpose of the research was not to precisely determine the properties of the tested headphones, but to select headphones that will be more suitable for the developed tester). The second reason was due to the many years of experience in the testing of the sound attenuation of hearing protectors (i.e., the high repeatability of responses to the test signal). The selected subjects also met the requirements of EN ISO 4869-1:2018 [3] (i.e., they had a pure-tone hearing threshold of no more than 15 dB for frequencies of 2000 Hz and below, and of no more than 25 dB for frequencies above 2000 Hz). The tests used the Norsonic NOR838 sound attenuation measurement system, which was controlled by a PC, a Rotel RMB-1075 power amplifier, and four JBL 4208 speaker sets. The test signal was pink noise filtered in 1/3 octave bands for a center frequency ranging from 125 Hz to 8000 Hz [3]. The sound attenuation measurements of the headphones were performed once for each frequency band. This is normal practice when measuring the sound attenuation of hearing protectors [3]. The order of testing for each subject was the same (i.e., first the measurement of the hearing threshold without headphones on and then the measurement with a particular set of headphones on). As stated above, because the subjects have a high repeatability of responses to the test signal, the order of measurement has little influence on the results.

**Figure 6:**
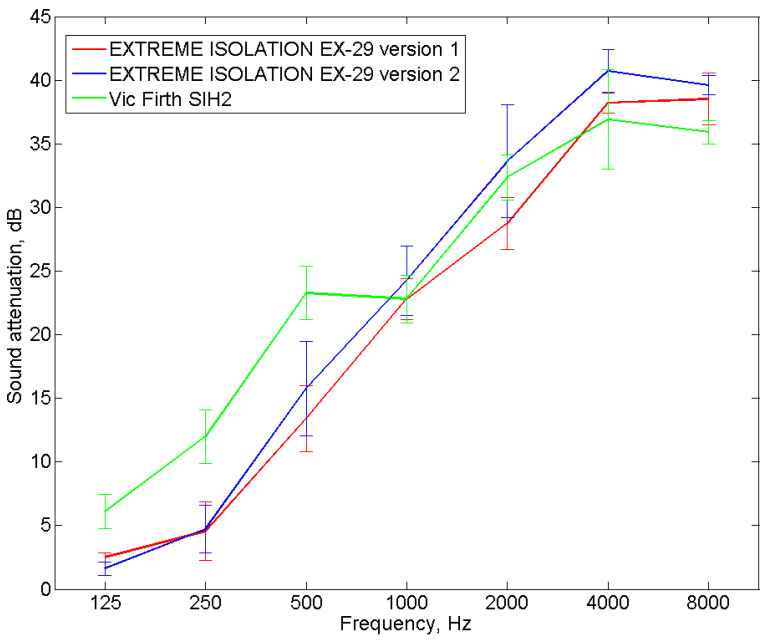
The mean value of the sound attenuation of the headphones.

The difference between the hearing thresholds determined with and without headphones represents the sound attenuation of a particular set of headphones. Figure 6 shows the mean value of the sound attenuation of the tested headphones.

The results presented in Figure 6 indicate that the Vic Firth SIH2 headphones will more intensively attenuate sound in the frequency range of 125–500 Hz than the Extreme Isolation EX-29 headphones. The standard deviations for the frequencies 125, 250, and 500 Hz were relatively small compared to the differences between the mean attenuations. However, in the remaining range of frequencies, the sound attenuation of the tested headphones was similar. It is important to note that in the case of Extreme Isolation EX-29 headphones, the sound attenuation values at the frequencies of 125 and 250 Hz were quite low and did not exceed 5 dB. Based on the conducted tests, it can therefore be concluded that due to higher sound attenuation values, the Vic Firth SIH2 headphones were selected for use in the tester to check the correct placement of the earplugs. The differences between the sound attenuation of individual headphones are most likely due to differences in their design including the tightness of the fit of the components of these headphones. In the frequency range of up to 500 Hz, selected physical parameters such as cup volume, cushion stiffness, and cushion contact area have a significant influence on sound attenuation.

### 3.2. Measurements of Sound Attenuation Performed with the Use of the Tester

**Figure 7:**
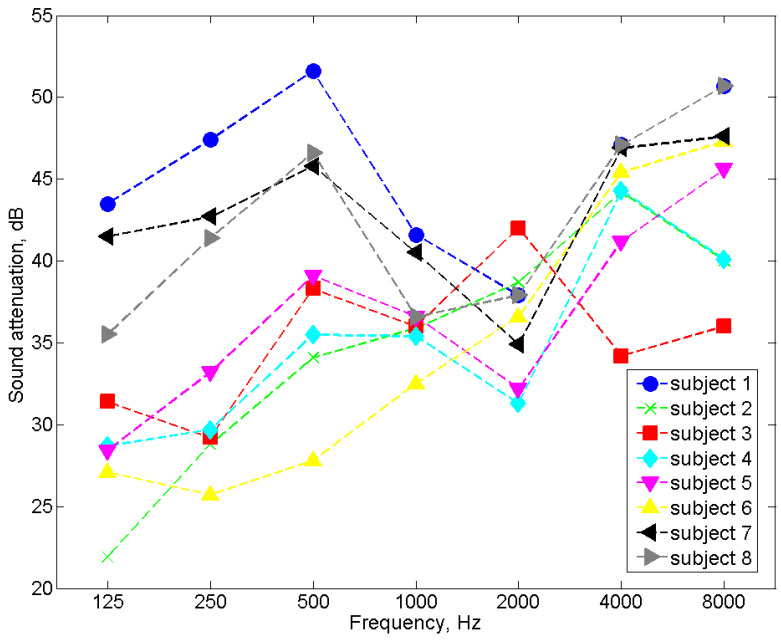
The sound attenuation of earplugs measured using the tester.

**Figure 8:**
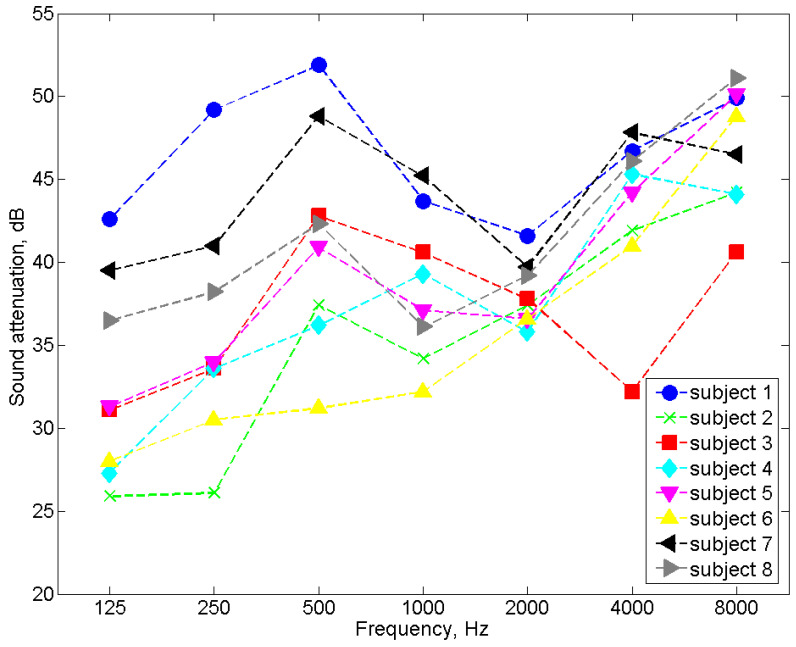
The sound attenuation of the earplugs measured using the Norsonic NOR838 system.

**Figure 9:**
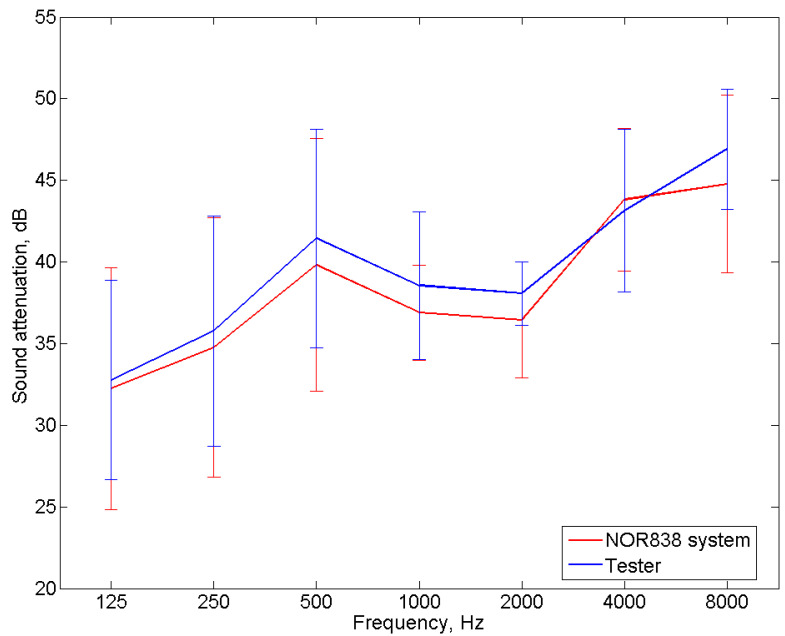
The mean sound attenuation values of the earplugs with the standard deviation measured using the tester and the Norsonic NOR838 system.

In order to verify the correct operation of the tester, measurements of the sound attenuation of the popular 3M 1100 model of earplugs were carried out using the tester. A total of eight subjects (five men and three women between the ages of 20 and 45) took part in the tests. As previously mentioned, 16 subjects are typically involved in sound attenuation tests. However, eight participants are also allowed (e.g., for product checks). In addition, for the study, we selected eight people who had a high repeatability of response to the test signal, and they were trained in placing earplugs in the ear canal. For the measurements with headphones, the selected subjects met the requirements of the EN ISO 4869-1:2018 standard [3] concerning the hearing threshold. The results of the sound attenuation measurement performed with the use of the tester were compared with the results of the sound attenuation measurements performed with the use of a reference system dedicated to this purpose (i.e., the Norsonic NOR838 system with the equipment described in Section 3.1). Comparative tests were carried out with the same people. To be independent of the influence of the method used to place the earplugs in the ear canal, after the insertion of the earplugs, measurements were taken successively on the tester and the Norsonic NOR838 system without replacing the earplugs. The tests were performed in the same order for all frequencies and across all subjects. As mentioned earlier, the participants had many years of experience in measuring sound attenuation and had a high repeatability of response to the test signal, so the order of measurement should not affect the results. As with the headphone sound attenuation measurements, the test signal was pink noise filtered in 1/3 octave bands for a center frequency ranging from 125 Hz to 8000 Hz. Figure 7 and Figure 8 present the results of the sound attenuation measurements of earplugs carried out with the use of the tester and the Norsonic NOR838 system as the reference system for individual subjects, respectively. The differences in sound attenuation values for individual subjects are presented in Table 1. On the other hand, the mean values of the sound attenuation of earplugs with the standard deviation are presented in Figure 9.

On the basis of the results of the sound attenuation measurements determined for individual participants using the developed tester (Figure 7) and the Norsonic NOR838 system (Figure 8), it could be observed that the differences between the sound attenuation values (Table 1) were not greater than 4.8 dB. According to the requirements of EN ISO 4869-1:2018 [3], the repeatability criterion for the measurement of the hearing threshold for the determination of sound attenuation is 6 dB. However, for the mean values, the differences between the sound attenuation measured with the tester and the Norsonic NOR838 system were 0.5, 1.0, 1.6, 1.7, 1.6, −0.7 and 2.1 dB for the frequencies of 125, 250, 500, 1000, 2000, 4000 and 8000 Hz, respectively. These values are fairly small and indicate the correct operation of the tester. The standard deviation values of the measurements conducted with the use of the NOR838 system ranged from 3 to 8 dB depending on the frequency, while the measurements realized with the use of the tester ranged from 2 to 7 dB. These high standard deviation values were mainly the result of a discrepancy in the shape of the subjects’ outer ear canal, which caused subjects to place the earplugs differently. Such standard deviation values and even higher values can be found in the user information for commonly used earplugs.

The statistical analysis showed that the mean sound attenuation values obtained by both devices did not have a statistically significant difference. The *p*-values (Student’s *t*-test) for individual frequencies are presented in Table 2. Before performing the Student’s *t*-test, the hypothesis that the data were characterized by a normal distribution (Lilliefors test) was verified, and the equality of the variance criterion was also verified.

In addition, traditional statistical analysis was supplemented with statistical analysis based on a Bayesian approach. Table 3 shows the *BF*_10_ coefficient calculated using the open-source JASP Bayesian interface [34]. Tests indicated anecdotal evidence for the hypothesis that the sound attenuation values obtained for both devices did not differ.

**Figure 10:**
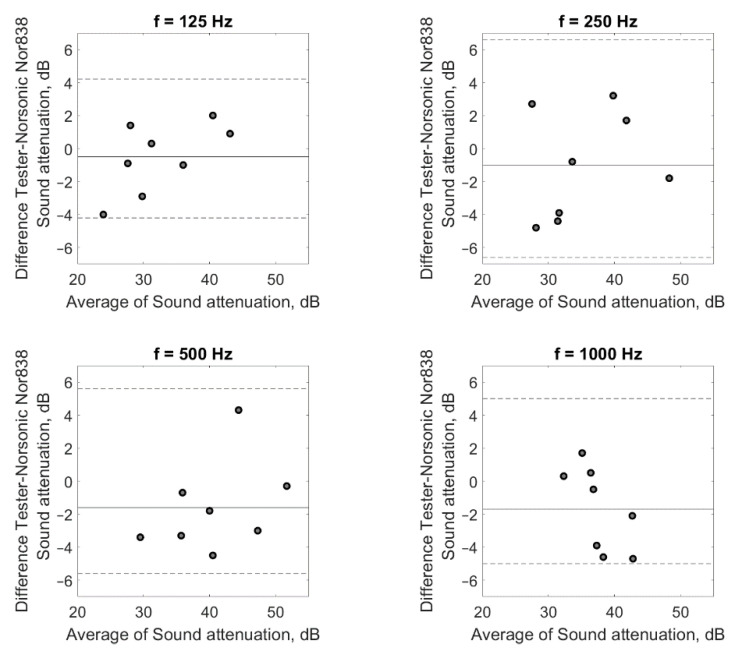

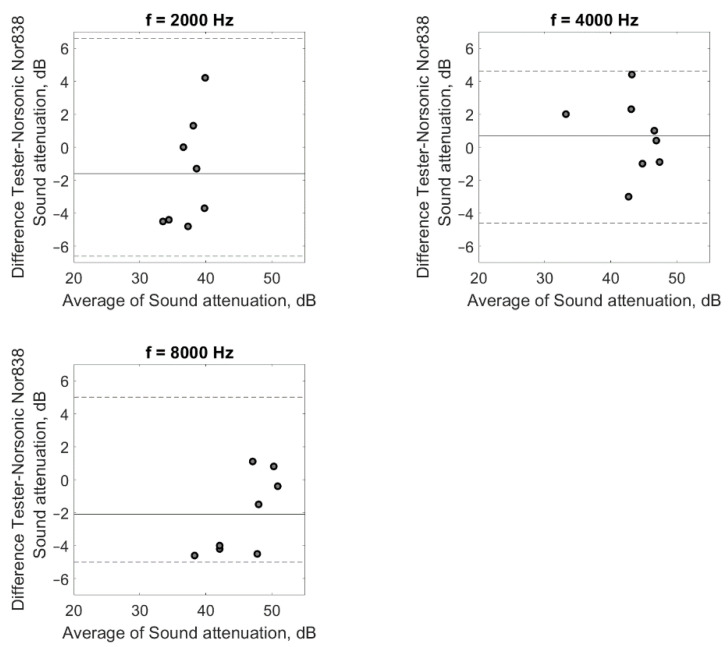
The Bland–Altman plots depicting the sound attenuation differences between the measurements conducted with the use of tester and the Norsonic NOR838 system at 125, 250, 500, 1000, 2000, 4000, and 8000 Hz. The solid line represents the mean value, the dashed lines represent ±2 standard deviations.

In addition to the statistical analysis, Bland–Altman plots were created for all frequencies to investigate the agreement between the two devices. The results in Figure 10 show that for all frequencies, the sound attenuation differences between the measurements conducted with the use of the tester and the Norsonic NOR838 system were between the lines denoting two standard deviations. This means an agreement between the sound attenuation values obtained for both devices.

The correct operation of the tester was proven by the test results obtained for individual subjects and the comparison of the mean sound attenuation values obtained using the developed tester and the Norsonic NOR838 system.

## 4. Discussion

It is possible in practice to establish that the earplugs properly attenuate noise only after checking whether their insertion into the ear canal is correct. For example, this is possible with specifically designed equipment. Several commercial devices are available on the market. These include, but are not limited to, FitCheck Solo (Michael and Associates, State College, PA, USA) [27], INTEGRAfit (Workplace Integra, Greensboro, NC, USA) [28], QuickFit (NIOSH, Washington, DC, USA) [29], VeriPRO (Honeywellt, Charlotte, USA) [30], E-A-Rfit (3M, Maplewood, MN, USA) [31], and SafetyMeter (Sonova Communications AG, Stäfa, Switzerland) [32]. The functioning of the first three solutions, which are listed in the same way as the described device, is based on the measurement of the hearing threshold of the person not wearing earplugs and wearing earplugs. In contrast to the tester developed and described in the manuscript, FitCheck Solo requires the use of a computer on which the software performs, among other things, the hearing threshold measurement function. The software is installed and a sound card in the computer is used. The measurement results are the PAR parameters, which are compared to the value of NRR determined on the basis of the measurement of sound attenuation according to ANSI S12.6-2016 [35]. The INTEGRAfit solution is used in a similar manner. However, INTEGRAfit requires the use of an iPad on which the corresponding application is installed. Similar to FitCheck Solo, the test result is compared to the NRR value. Similar to the tester developed by the authors, QuickFit is independent of external devices such as a computer or a tablet. This device also relies on the measurement of the hearing threshold in order to determine the attenuation of the earplugs, but in a very simplified manner. The test involves setting the level of the test signal at its hearing threshold. When the earplugs are inserted, the device generates the same test signal, but with a sound level 15 dB higher. If no test signal is heard, the earplugs are positioned correctly. The device itself is composed of a cup of earmuffs in which an mp3 player is located, and a loudspeaker, which is how a test signal is generated. The tester presented in this study, in contrast to QuickFit, allows for accurate measurements in a wider (125 to 8000 Hz) frequency range. In the case of VeriPRO, a method of checking the correct placement of the earplugs, other than in the device in question, was used. This method is based on the comparison of the volume in both ears of the subject being tested. The subject tested first equalizes the volume of the test signal without earplugs, then with one earplug inserted in the ear canal, so that it is possible to check that this insert is correctly positioned, and then with the two earplugs inserted in the ear canal. In contrast to the tester presented in this study, VeriPRO is quite advanced and expensive. It consists of an audio processor in a separate module, dedicated headphones, and computer software. The E-A-Rfit device uses the MIRE (microphone in real ear) method to determine if the earplugs are correctly inserted into the ear canal. The device consists of a speaker equipped with a digital signal processor, microphones (placed in the tested earplugs and outside the earplugs), and software. A test signal is generated from the speaker, the sound pressure level of which is measured both under and outside the earplugs. The measured difference between the values of the sound pressure level represents the attenuation determining whether or not the earplugs are correctly inserted. The SafetyMeter device operates in a similar manner. The device consists of a module (sound card) connected to a computer. The module is connected to headphones that generate a test signal and two microphones that are inserted into the earplugs. Both the E-A-Rfit and SafetyMeter devices only enable the earplugs of the manufacturers of the individual devices to be tested. The developed tester differs from most commercial devices in that it can operate integrally without being connected to a computer. The tester enables measurements to be carried out over a wide range of frequencies and can be used for all types of earplugs. Table 4 summarizes the basic characteristics of the tester compared to the commercial equipment that is available on the market.

The basic characteristics of several devices that are available on the market and the tester developed in this work were compared as devices that make it possible to evaluate insert attenuation in a simplified manner. The evaluation of the properties of the developed tester, on the other hand, was carried out in this work in relation to a reference device and treated as a standard-setting device for measuring sound attenuation (i.e., enabling precise measurements according to the requirements of the standard).

The developed tester can be used by people with normal hearing. Moreover, there is no contraindication for the tester to be used by employees with moderate hearing loss. Studies presented in the literature [7,36] indicate that for such a group of people, the results of sound attenuation measurements using the REAT method will not differ significantly from the results of measurements performed on people with normal hearing. This is due to the principle of sound attenuation measurement (i.e., the difference between the hearing thresholds of a person with and without a hearing protector is counted). Therefore, moderate hearing loss should also have no effect on the accuracy of the sound attenuation measurement of the developed tester. The situation may be different for people with severe hearing loss (i.e., the hearing threshold may not change despite wearing a hearing protection device). However, such people should not be allowed to work in noisy environments, so the problem of using earplugs does not apply to them.

## 5. Conclusions

The presented tester was made of low-cost components including a Raspberry Pi Zero single-board computer, an independent digital-to-analog converter, a headphone amplifier, headphones, an LCD with a touch panel and a battery. The tester cannot improve the position of the earplugs in the ear canal, but allows one to check if these earplugs attenuate noise appropriately. If insufficient attenuation is observed, the user can correct the insertion of the earplugs.

In order to verify the correct operation of the tester, measurements of the sound attenuation of the earplugs were carried out using the tester. The results of the sound attenuation measurement performed with the use of the tester were compared with the results of the sound attenuation measurements performed with the use of the Norsonic NOR838 system dedicated to this purpose.

The differences in the results of the measurement of the sound attenuation measured for individual participants using the developed tester and reference system were not greater than 4.8 dB. However, taking into account the mean values, the difference between the sound attenuation measured with the tester and the Norsonic NOR838 system ranged from 0.5 dB for the frequency of 125 Hz to 2.1 dB for the frequency of 8000 Hz. These values are fairly small and indicate the correct operation of the tester. As a result, the developed tester is considered to be a device with which the obtained sound attenuation values will be reliable so that it can also be used as a device to assess the correct placement of earplugs in the ear canal.

## Figures and Tables

**Table 1:** The difference in the sound attenuation values for individual subjects.

Frequency, Hz		125	250	500	1000	2000	4000	8000
Sound attenuation difference, dB	Subject 1	0.9	−1.8	−0.3	−2.1	−3.7	0.4	0.8
	Subject 2	−4.0	2.7	−3.3	1.7	1.3	2.3	−4.2
	Subject 3	0.3	−4.4	−4.5	−4.6	4.2	2.0	−4.6
	Subject 4	1.4	−3.9	−0.7	−3.9	−4.5	−1.0	−4.0
	Subject 5	−2.9	−0.8	−1.8	−0.5	−4.4	−3.0	−4.5
	Subject 6	−0.9	−4.8	−3.4	0.3	0.0	4.4	−1.5
	Subject 7	2.0	1.7	−3.0	−4.7	−4.8	−0.9	1.1
	Subject 8	−1.0	3.2	4.3	0.5	−1.3	1.0	−0.4

**Table 2:** The results of the verification of whether the mean sound attenuation values obtained using the tester and the Norsonic NOR838 system had a statistically significant difference.

Frequency, Hz	125	250	500	1000	2000	4000	8000
*p*-value	0.8798	0.7926	0.6644	0.3047	0.2713	0.7820	0.3723

**Table 3:** The results of the verification with the use of Bayesian approach of whether the mean sound attenuation values obtained using the tester and the Norsonic NOR838 system did not differ.

Frequency, Hz	125	250	500	1000	2000	4000	8000
*BF*_10_	0.4311	0.4384	0.4565	0.5533	0.6667	0.439	0.5714

**Table 4:** The basic characteristics of the tester compared to the commercial equipment that is available on the market.

Device	Measurement Method	Control	Measured Frequencies	Application
Tester presented in this study	Audiometry-based	Complete instrument	125, 250, 500, 1000, 2000, 4000, 8000	All earplugs.
FitCheck Solo	Audiometry-based	PC-based	125, 250, 500, 1000, 2000, 4000, 8000	All earplugs.
INTEGRAfit	Audiometry-based	iPad-based	500, 1000, 2000	All earplugs.
QuickFit	Audiometry-based	Complete instrument	1000	All earplugs.
VeriPRO	Loudness balancing	PC-based	125, 250, 500, 1000, 2000, 4000	All earplugs.
E-A-Rfit	MIRE *	PC-based	125, 250, 500, 1000, 2000, 4000, 8000	Only for earplugs from the device manufacturer
SafetyMeter	MIRE	PC-based	125, 250, 500, 1000, 2000, 4000, 8000	Only for earplugs from the device manufacturer

* MIRE—microphone in real ear.

## Data Availability

All of the data are stored digitally by the researchers.
